# Gut microbiota and metabolite profiles in HBV cirrhosis with persistent liver enzyme abnormalities

**DOI:** 10.3389/fmicb.2025.1645023

**Published:** 2025-10-10

**Authors:** Huan He, Ruixue Zou, Xinyi Liao, Yinghua Lan

**Affiliations:** Key Laboratory of Molecular Biology for Infectious Diseases (Ministry of Education), Department of Infectious Diseases, Institute for Viral Hepatitis, the Second Affiliated Hospital, Chongqing Medical University, Chongqing, China

**Keywords:** hepatitis B virus, liver cirrhosis, HBV-DNA, microbiome, metabolome, liver enzymes

## Abstract

**Background and aims:**

Dysbiosis of gut microbiota and metabolic disturbances are implicated in the progression of hepatitis B virus (HBV)-related cirrhosis. However, the mechanisms underlying these associations, particularly in patients with undetectable HBV-DNA following antiviral therapy but persistent liver enzyme abnormalities, remain poorly understood. This study aimed to characterize the gut microbiota and metabolic profiles in this patient population to elucidate potential mechanisms and identify biomarkers.

**Materials and methods:**

Sixty-five patients with HBV-related cirrhosis were enrolled. Fecal samples were analyzed using 16S rRNA gene sequencing and untargeted liquid chromatography-mass spectrometry (LC–MS) to assess gut microbiota composition and metabolite profiles. Correlation analyses and multivariate statistical approaches were employed to explore relationships between microbiota, metabolites, and clinical parameters.

**Results:**

Significant gut microbiota dysbiosis was observed in HBV-related cirrhosis patients. Comparative analysis identified 15 differentially abundant genera and 431 metabolites between patients with normal and abnormal liver enzyme levels. Notably, *Blautia* abundance was positively correlated with ursocholic acid (UCA), which was significantly reduced in patients with abnormal liver enzymes. UCA levels were inversely associated with AST, TBIL, and ALP, suggesting its potential role in modulating liver enzyme activity.

**Conclusion:**

These findings highlight the gut-liver axis as a driver of persistent liver injury and identify microbial (e.g., Blautia) and metabolic (e.g., UCA, TDC) signatures as potential biomarkers for HBV cirrhosis.

## Introduction

Hepatitis B virus (HBV) infection poses a persistent global health burden, driving the progression of chronic liver disease, cirrhosis, and hepatocellular carcinoma (HCC) (Hsu et al., 2023). In HBV-related cirrhosis, sustained liver enzyme abnormalities—even after achieving undetectable HBV-DNA through antiviral therapy—signal ongoing hepatic injury, accelerating fibrosis progression and increasing risks of decompensation and HCC (Boursier et al., 2016). These abnormalities may further reflect impaired hepatic metabolic functions, including disrupted protein synthesis and detoxification pathways, thereby compounding clinical morbidity (Albillos et al., 2020).

Accumulating evidence has characterized gut microbiome (GM) dysbiosis in HBV-related liver disease, with microbial alterations directly linked to disease severity and clinical outcomes. Chronic HBV infection exhibits reduced microbial α-diversity and enriched pro-inflammatory taxa (e.g., Enterobacteriaceae), correlating with hepatic inflammation and fibrosis progression (Shen et al., 2023). Longitudinal analyses further reveal dynamic GM shifts during disease evolution, where Streptococcus- and Veillonella-dominated profiles predict cirrhosis development (Chen et al., 2020). In decompensated cirrhosis, GM signatures (e.g., depletion of Lachnospiraceae) serve as prognostic markers for acute-on-chronic liver failure (Wang et al., 2021), while metabolomic perturbations—particularly disrupted bile acid and aromatic amino acid metabolism—differentiate disease stages (Shi et al., 2025). Collectively, these findings establish the GM as a key regulator of HBV pathogenesis, though the microbial-metabolite drivers of persistent inflammation in virologically suppressed patients remain mechanistically undefined.

Emerging evidence highlights the gut-liver axis as a pivotal mediator of liver disease pathogenesis. This bidirectional network integrates metabolic, immune, and neuroendocrine crosstalk via portal circulation and biliary excretion (Tripathi et al., 2018). A functionally intact intestinal barrier—comprising chemical (e.g., antimicrobial peptides), physical (e.g., epithelial tight junctions), and immunological (e.g., gut-associated lymphoid tissue) components—prevents microbial translocation. However, cirrhosis-induced dysbiosis disrupts this equilibrium, favoring pathobionts (e.g., *Enterobacteriaceae*) over commensals (e.g., *Bifidobacterium, Lactobacillus*), increasing intestinal permeability, and triggering hepatic inflammation through pathogen-associated molecular pattern (PAMP) release (Odenwald and Turner, 2017; Shu et al., 2022).

While 16S rRNA sequencing has delineated taxonomic shifts in cirrhosis, functional insights into host-microbiota interplay require metabolomic integration. Microbial-derived metabolites—including bile acids, short-chain fatty acids, and trimethylamine—directly modulate hepatic inflammation, fibrosis, and metabolic reprogramming (Sun et al., 2021). Fecal metabolomics thus bridges the gap between microbial composition and disease mechanisms, offering translational opportunities for therapeutic targeting.

Notably, a subset of HBV-related cirrhosis patients with virological suppression (undetectable HBV-DNA) exhibits persistent liver enzyme elevations, suggesting unresolved gut-liver axis dysregulation. However, the microbial and metabolic signatures underlying this phenotype remain uncharacterized. Here, we employed 16S rRNA gene sequencing and untargeted liquid chromatography-mass spectrometry (LC–MS) to profile gut microbiota and metabolites in this high-risk population, aiming to uncover mechanistic drivers and potential therapeutic targets.

## Materials and methods

### Patients and study design

This prospective cohort study prospectively enrolled 78 patients with HBV-related cirrhosis from the Second Affiliated Hospital of Chongqing Medical University between May 2023 and July 2024. Following propensity score matching to minimize confounding factors, 65 patients were stratified into two groups: normal liver enzymes (*n* = 37) and persistent abnormal liver enzymes (*n* = 28). Non-targeted metabolomics profiling was subsequently performed on plasma samples from 55 patients within this matched cohort (32 normal enzymes, 23 persistent abnormalities). Inclusion criteria required: (1) HBsAg positivity ≥6 months with confirmed cirrhosis diagnosis (histopathological evidence of diffuse fibrosis and regenerative nodules, or ≥2 clinical criteria: esophageal/gastric varices on endoscopy, imaging-confirmed cirrhosis, liver stiffness >13 kPa, impaired synthetic function, or hypersplenism); (2) Sustained virological suppression (serum HBV-DNA < 20 IU/mL) for ≥1 year under antiviral therapy; (3) Persistent elevation of liver enzymes (ALT, AST, GGT, ALP) despite virological control; (4) Child-Pugh class A/B. Exclusion criteria included: (1) Metabolic disorders (uncontrolled diabetes/dyslipidemia, abnormal urinalysis/fecal occult blood); (2) Coexisting liver pathologies (autoimmune hepatitis, HCC, NAFLD); (3) Recent use of antibiotics/probiotics (≤8 weeks) or immunosuppressants; (4) Alcohol consumption or hepatotoxic drug exposure. The study protocol complied with the Declaration of Helsinki (1975) and was approved by the Ethics Committee of the Second Affiliated Hospital of Chongqing Medical University (Approval No. KY2024-075). All participants provided written informed consent.

### DNA extraction and 16S rRNA gene amplicon sequencing and analysis

Fecal samples (50–200 mg) were aseptically collected from the stool interior (avoiding surface contamination and urine), flash-frozen within 2 h, and stored at −80 °C. Subsequent DNA extraction was followed by 16S rRNA gene amplification (V3–V4 regions), amplicon pooling, purification, and library preparation. Libraries were quantified using Qubit and qPCR, with qualified libraries subjected to Illumina sequencing. After bioinformatic quality control, denoising and Amplicon Sequence Variant (ASV) inference were performed in QIIME2 (DADA2 module) with taxonomic assignment against the SILVA 138.1 database. Alpha diversity (observed ASVs, Shannon, Simpson, Chao1, Goods_coverage, dominance,) was calculated in QIIME2; rarefaction curves (generated in R using plyr) confirmed sequencing depth adequacy. Beta diversity analysis employed Bray-Curtis distances; PCoA visualization (Bray-Curtis) used R. Inter-group differences were assessed via *t*-tests. Similarity Percentage (SIMPER) analysis was performed to identify genera contributing most to inter-group dissimilarities (Bray-Curtis index). The top 10 genera accounting for >70% cumulative dissimilarity were visualized.

### Fecal metabolome profiling and analysis

The raw data were firstly converted to mzXML format by MSConvert in ProteoWizard software package (Rasmussen et al., 2022) and processed using R XCMS (v3.12.0) for feature detection (Navarro-Reig et al., 2015), retention time correction and alignment. Key parameters settings were set as follows: ppm = 15, peakwidth = c (5, 30), mzdiff = 0.01, method = centWave. The batch effect was then eliminated by correcting the data based on QC samples. Metabolites with RSD > 30% in QC samples were filtered and then used for subsequent data analysis.

The metabolites were identified by accuracy mass and MS/MS data which were matched with HMDB^1^ (Wishart et al., 2007), KEGG^2^ (Kanehisa and Goto, 2000), the metabolite database bulid by Panomix Biomedical Tech Co., Ltd. (Shuzhou, China). The molecular weight of metabolites was determined according to the m/z (mass-to-charge ratio) of parent ions in MS data. Molecular formula was predicted by ppm (parts per million) and adduct ion, and then matched with the database. At the same time, the MS/MS data from quantitative table of MS/MS data, were matched with the fragment ions and other information of each metabolite in the database, so as to realize the MS/MS identification of metabolites.

Two different multivariate statistical analysis models, unsupervised and supervised, were applied to discriminate the groups (OPLS-DA) by R ropls package. The statistical significance of *P* value was obtained by statistical test between groups. Finally, combined with *P* value, VIP (OPLS-DA variable projection importance) and FC (multiple of difference between groups) to screen biomarker metabolites. By default, when *P* value < 0.05 and VIP value > 1, we think that metabolite were considered to have significant differential expression.

### Statistical analysis

Data analysis was performed using SPSS 27.0 and R. Continuous variables are expressed as mean ± standard deviation (SD) for normally distributed data or median (interquartile range, IQR) for non-normally distributed data. Normality was assessed using Shapiro–Wilk tests. Group comparisons were conducted as follows: (1) normally distributed variables were analyzed with two-tailed independent Student’s *t*-tests; (2) non-normally distributed variables were evaluated using Mann–Whitney U tests; (3) categorical variables were compared via chi-square tests. Spearman’s rank correlation analysis was applied to assess associations between microbial genera, metabolites, and clinical parameters, correlation analyses (*n* = 1,000 bootstrap iterations) were performed to assess robustness. To account for multiple testing, the Benjamini-Hochberg false discovery rate (FDR) correction was applied, with an adjusted *p* < 0.05 defining statistical significance.

## Results

### Patient backgrounds

The study cohort included 37 virologically suppressed patients with normal liver enzymes and 28 patients exhibiting persistent liver enzyme abnormalities. Within the abnormal enzyme group, 13 cases were histopathologically confirmed, while 15 met composite clinical/imaging criteria; by contrast, only 8 cases in the normal enzyme group underwent histopathological assessment.

Baseline demographic and clinical characteristics—including age, BMI, sex distribution, prothrombin time activity (PTA), alpha-fetoprotein (AFP) levels, and Child-Pugh classification—demonstrated no significant intergroup differences (all *p* > 0.05), indicating well-matched cohorts. Notably, the abnormal enzyme group displayed significantly elevated serum biomarkers of hepatic injury compared to controls: Aspartate aminotransferase (AST; *p* = 0.001), alanine aminotransferase (ALT; *p* = 0.044), gamma-glutamyl transferase (GGT; *p* < 0.001), alkaline phosphatase (ALP; *p* < 0.001), total bilirubin (TBIL; *p* = 0.009).

This biochemical signature (summarized in Table 1) strongly suggests ongoing hepatocellular damage despite achieved virological suppression, highlighting a clinically relevant dissociation between viral control and hepatic homeostasis.

**Table 1:** Characteristics of studied groups.

Demographic characteristics	Abnormal group (*n* = 28)	Normal group (*n* = 37)	*p*-value
Male (number)	19 (67.9%)	25 (67.6%)	0.980
Age (year)	55.18 ± 9.68	56 ± 8.239	0.713
BMI (kg/m^2^)	23.2261 ± 2.52302	23.4008 ± 2.91473	0.801
AST (U/L)	53.71 ± 37.697	27.62 ± 7.682	0.001
ALT (U/L)	55.61 ± 81.702	22.92 ± 7.701	0.044
ALP (U/L)	120.04 ± 43.113	72.22 ± 20.096	<**0.001**
GGT (U/L)	92.82 ± 66.974	28.03 ± 13.841	<0.001
TBIL (umol/L)	35.636 ± 29.2612	19.532 ± 11.1301	0.009
TBA (umol/L)	85.968 ± 148.5371	34.276 ± 88.5291	0.11
PTA (%)	80.19 ± 17.817	71.54 ± 22.948	0.092
AFP (ng/mL)	5.6518 ± 4.07204	3.99642 ± 2.12418	0.058
INR	1.2729 ± 0.3319	1.1722 ± 0.17003	0.092
Child-Pugh score	6.32 ± 1.182	6.14 ± 1.182	0.573
Compensated stage, *n* (%)	21 (75%)	27 (73%)	0.854

Data are presented in terms of the mean ± standard deviation. Bold font: *p* value < 0.05, *p < 0.05, **p < 0.01, ***p < 0.001.

AST, aspartate transaminase; ALT, alanine aminotransferase; ALP, alkaline phosphatase; GGT, gamma-glutamyl transferase; TBIL, total bilirubin; TBA, total bile acids; PTA, prothrombin activity; AFP, alpha-fetoprotein; INR, international normalized ratio.

### Gut microbial diversity profiling

**Figure 1:**
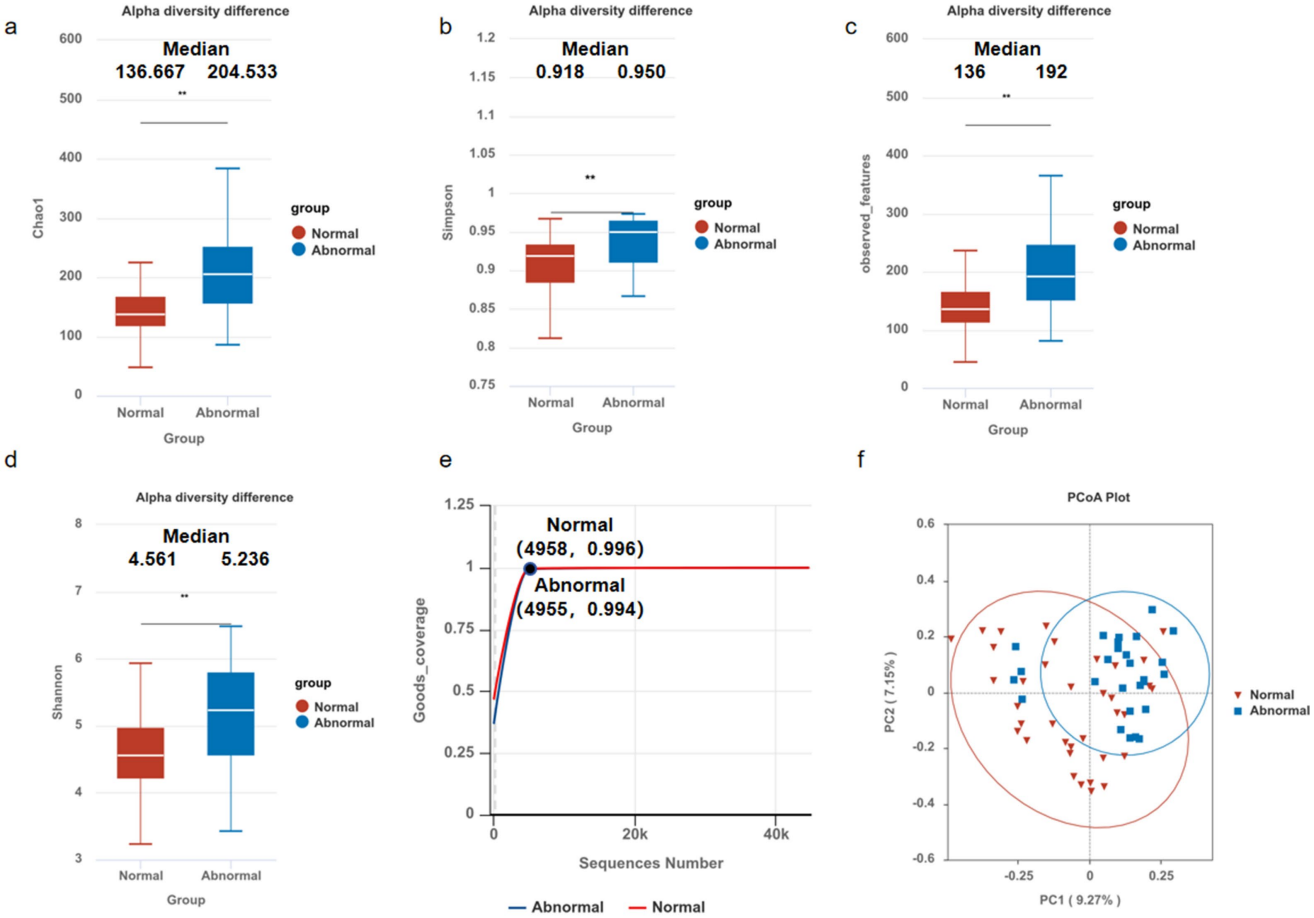
Microbial community diversity in hepatitis B-related liver cirrhosis. The alpha diversities of the gut microbiota between normal and abnormal groups were analyzed using Student’s *t*-test, showing significant differences in: **(a)** Chao1 index (136.667VS204.533), *p* < 0.05; **(b)** Simpson index (0.918VS0.950), *p* < 0.05; **(c)** observed features (136VS192), *p* < 0.05; **(d)** Shannon diversity (4.561VS5.236), *p* < 0.05; compared to those with normal liver enzyme. Results remained robust despite comparable sequencing depth (Good’s coverage, **e**). Boxplots represent median (center line), IQR (box), and 1.5 × IQR (whiskers). Beta diversities of the gut microbiota in patients between normal and abnormal groups were analyzed via PCoA **(f)**. The PCoA plot of Bray-Curtis dissimilarities illustrates the relationships and distances between microbial communities in different groups (*R* = 0.177, *p* = 0.0049), *p < 0.05, **p < 0.01, ***p < 0.001.

Contrary to the characteristic decline in microbial diversity observed in advanced liver disease, our analysis of cirrhotic patients with undetectable HBV demonstrated a paradoxical increase in α-diversity among subjects with persistent liver enzyme abnormalities compared to those with normal liver function. Specifically, the abnormal enzyme group exhibited significantly elevated microbial richness (Chao1 index, *p* < 0.05; Figure 1a) and evenness (Simpson index, *p* < 0.05; Figure 1b), corroborated by higher observed features (*p* < 0.05; Figure 1c) and Shannon diversity (*p* < 0.05; Figure 1d), despite comparable sequencing depth (Good’s coverage > 99%; Figure 1e).

β-Diversity analysis (Bray-Curtis PCoA) revealed significant compositional divergence between groups (ANOSIM *R* = 0.177, *p* = 0.0049; Figure 1f), confirming distinct microbial clustering in the abnormal enzyme cohort. Notably, these dysbiotic patterns persisted despite prolonged antiviral therapy and sustained virological suppression (HBV-DNA undetectable), suggesting that gut microbiota alterations may contribute to residual hepatic inflammation independently of active viral replication.

### Taxonomic profiling and dysbiosis patterns

**Figure 2:**
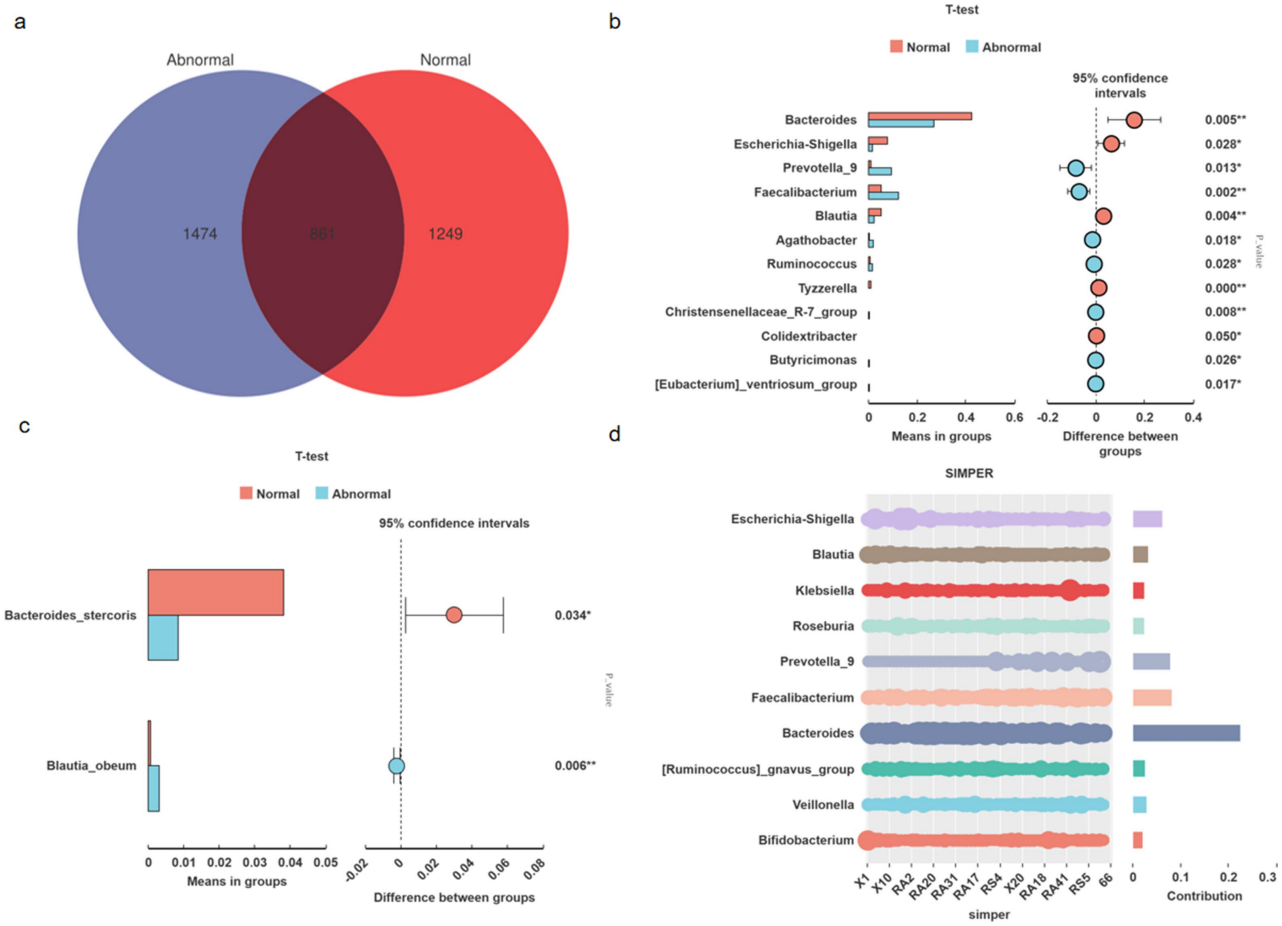
Microbial community structure and differential taxa between abnormal and normal groups. Venn diagram illustrating unique and shared amplicon sequence variants (ASVs) between groups (Abnormal: 1,474; Normal: 1,249; shared: 861) **(a)**. Genus-level taxonomic differences (*t*-test, FDR-corrected *p* < 0.05). Bar heights indicate relative abundance (%) **(b)**. Species-level differential abundance analysis (*t*-test, FDR-corrected *p* < 0.05). Bar heights indicate relative abundance (%) **(c)**. Key genera driving dissimilarities (SIMPER analysis). Bar heights show contribution to Bray–Curtis dissimilarity (%); cumulative line indicates explained dissimilarity **(d)**.

Microbiota analysis of 65 fecal samples revealed distinct amplicon sequence variant (ASV) distributions between groups, with the abnormal group and normal controls exhibiting 1,474 and 1,249 unique ASVs, respectively, alongside 861 shared ASVs (Figure 2a).

Genus-level comparative analysis identified significant intergroup differences (Figure 2b). The NORMAL group demonstrated enriched abundances of *Bacteroides* (*p* = 0.005), *Blautia* (*p* = 0.004), *Escherichia-Shigella* (*p* = 0.028), *Tyzzerella* (*p* < 0.001), and *Colidextribacter* (*p* = 0.050). Conversely, the ABNORMAL group showed elevated levels of *Faecalibacterium* (*p* = 0.002), *Christensenellaceae_R-7_group* (*p* = 0.008), *Prevotella_9* (*p* = 0.013), *Agathobacter* (*p* = 0.018), *Ruminococcus* (*p* = 0.028), *Butyricimonas* (*p* = 0.027), and *[Eubacterium]_ventriosum_group* (*p* = 0.017). Species-level analysis further revealed higher *Bacteroides stercoris* (*p* = 0.034) and lower *Blautia obeum* (*p* = 0.006) abundances in normal versus abnormal subjects (Figure 2c).

SIMPER analysis pinpointed the top 10 genera contributing to intergroup dissimilarities, with *Bacteroides, Faecalibacterium*, and *Prevotella_9* collectively accounting for >35% of compositional variation. These findings align with established mechanistic evidence: B*acteroides* enrichment promotes intestinal barrier integrity via acetate metabolism (Bajaj et al., 2012); F*aecalibacterium* depletion (a key butyrate producer) inversely correlates with hepatic inflammation severity (Oh et al., 2020); P*revotella_9* overabundance may potentiate lipopolysaccharide translocation, triggering TLR4-mediated hepatocyte injury (Qin et al., 2014).

Collectively, these taxonomically resolved functional shifts define a microbial paradigm for persistent liver enzyme abnormalities in virologically controlled HBV-related cirrhosis, suggesting that metabolic dysfunction—rather than mere compositional changes—drives clinical outcomes (Figure 2d).

### Fecal metabolomic landscape in HBV-related cirrhosis

#### Untargeted metabolomics reveals microbiota-driven metabolic reprogramming

**Figure 3:**
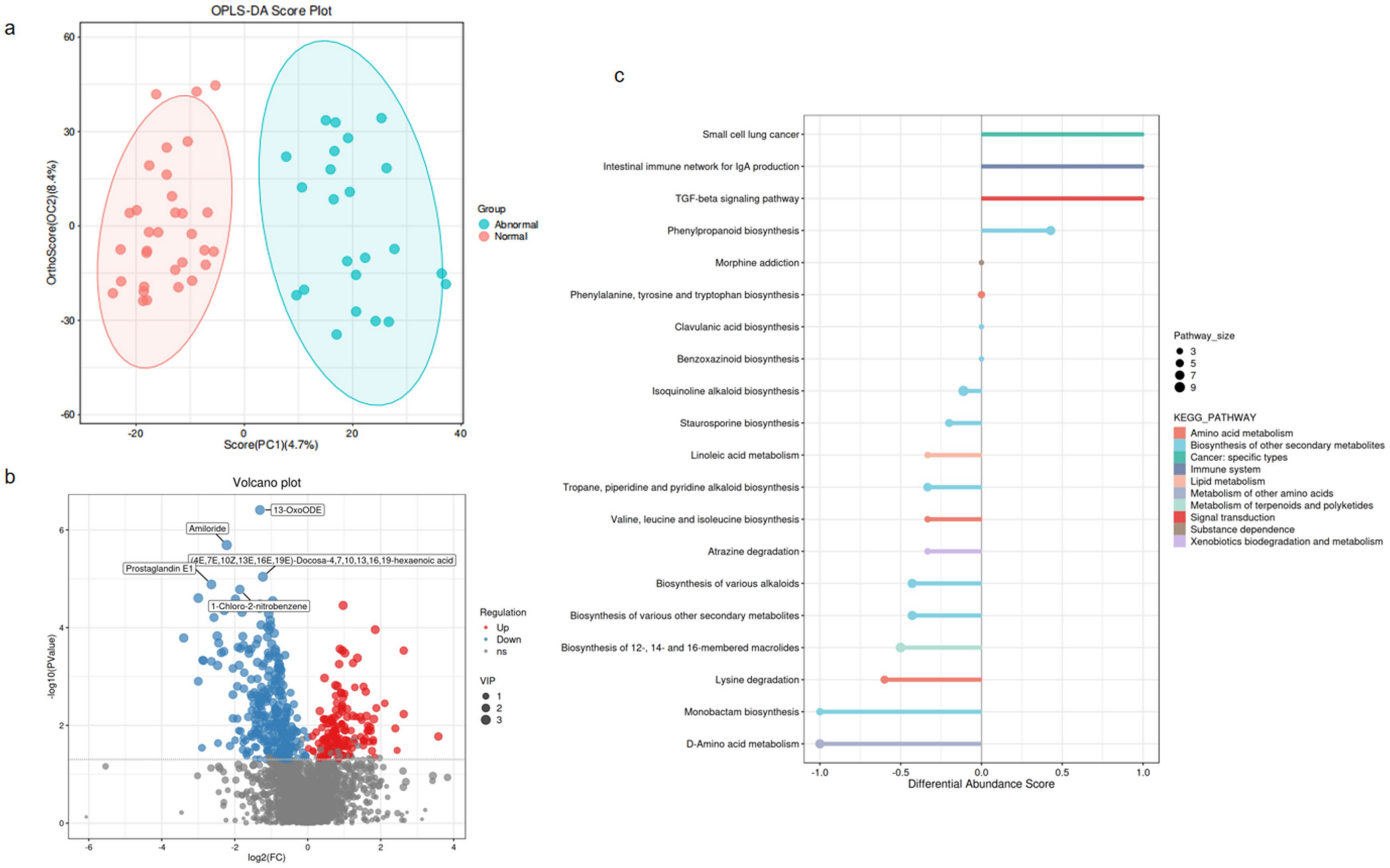
Overall alteration of fecal metabolites in hepatitis B-related liver cirrhosis. **(a)** OPLS-DA score plot (Abnormal vs. Normal). Ellipses represent 95% confidence intervals. **(b)** Volcano plot of differential metabolites (Normal vs. Abnormal). Thresholds: |log₂FC| > 1& FDR-adjusted *p* < 0.05. Red points: significantly upregulated metabolites; Blue: downregulated. **(c)** KEGG pathway enrichment (DA-score). Dot size: Pathway_size (number of metabolites mapped to pathway); Color: −log₁₀(FDR-adjusted *p*-value); Y-axis: Pathway impact score. Top 20 significantly enriched pathways shown (FDR < 0.05).

Untargeted metabolomic profiling was conducted on 55 patients with HBV-related cirrhosis (normal liver enzymes: *n* = 32; persistent abnormalities: *n* = 23) to elucidate gut microbiota-host metabolic interactions. Orthogonal partial least squares-discriminant analysis (OPLS-DA) revealed clear group separation (Figure 3a), confirming distinct metabolic remodeling in the abnormal cohort.

Among 2,823 annotated metabolites, 431 exhibited significant differential abundance (false discovery rate [FDR]-adjusted *p* < 0.05, variable importance in projection [VIP] > 1; Figure 3b; Supplementary Table S1). These dysregulated metabolites were primarily enriched in hepatic injury-associated pathways (Figure 3c), including: Amino acid metabolism (e.g., phenylalanine/tyrosine); Lipid metabolism (e.g., bile acid biosynthesis); Xenobiotics biodegradation (e.g., cytochrome P450); Immune system regulation (e.g., histidine-derived mediators); Cancer-related pathways (e.g., choline metabolism in hepatocellular carcinoma); Secondary metabolite biosynthesis (terpenoids/polyketides) and neuroactive substance dependence pathways were also perturbed, suggesting multifaceted microbiota-host crosstalk in HBV-related cirrhosis.

Notably, microbiota-mediated perturbations—particularly bile acid dysregulation and reduced immunomodulatory tryptophan catabolites—emerged as potential drivers of persistent hepatic inflammation and fibrosis in virologically suppressed HBV cirrhosis.

**Figure 4:**
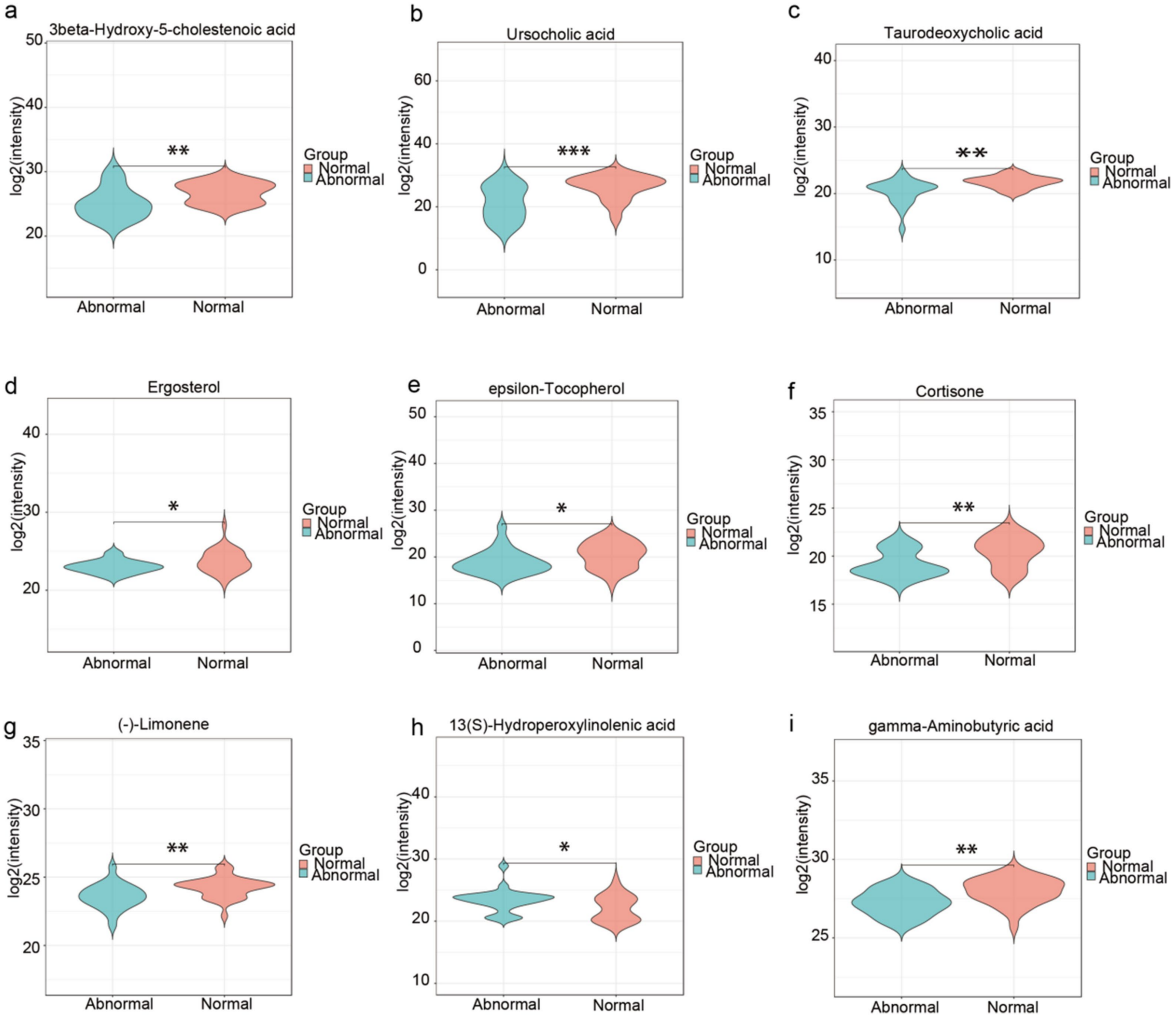
High-confidence fecal metabolites discriminating abnormal liver function in HBV-related cirrhosis. **(a–i)** Boxplots of 9 clinically relevant metabolites* (selected from 52 Level-1 verified biomarkers). Statistical significance: *p < 0.05, **p < 0.01, ***p < 0.001. Metabolite grouping by pathological mechanism, Bile acid dysregulation: 3-β-Hydroxy-5-cholestenoic acid, Ursocholic acid (UCA), Taurodeoxycholic acid (TDC), Micronutrient depletion: Ergosterol, ε-Tocopherol, Neuroendocrine disruption: Cortisol, Gamma-aminobutyric acid (GABA). Gut-liver axis modulation: (−)-Limonene, 13(S)-Hydroperoxylinolenic acid.

Untargeted metabolomics profiling identified 2,823 metabolites in total. Following variable importance in projection (VIP) screening (VIP > 1) and statistical thresholding (*p* < 0.05), 431 differentially abundant metabolites were initially selected. Applying stringent Level-1 verification criteria—requiring matched MS1 and MS2 spectra to authenticated standards (highest confidence level) and exclusion of unclassified metabolites—yielded 52 high-confidence discriminant metabolites (detailed in Supplementary Table S1). From this refined dataset, we prioritized 9 metabolites with established clinical relevance to liver pathology for further mechanistic investigation.” Multiple metabolites demonstrated significant intergroup disparities, with the abnormal group exhibiting marked alterations compared to the normal group in the following metabolites, suggesting their mechanistic relevance to hepatic dysfunction:bile acid dysregulation [e.g., reduced 3-β-Hydroxy-5-cholestenoic acid (Wilmanski et al., 2019), ursocholic acid (UCA) (Paumgartner and Beuers, 2002), and taurodeoxycholic acid (TDC) (Cai et al., 2022), enriched Deoxycholic Acid Glycine Conjugate (DCA-Gly)], micronutrient and antioxidant depletion (e.g., reduced ergosterol and *ε*-tocopherol) (Luo et al., 2022), neuroendocrine and immunometabolic disruption [e.g., reduced cortisol (Polyzos and Targher, 2024) and gamma-aminobutyric acid (Montagnese et al., 2021)] and gut-liver axis modulation (e.g., decreased (−)-limonene and increased 13(S)-hydroperoxylinolenic acid). These findings underscore the pathogenic contributions of bile acid dysregulation, oxidative stress, and gut-liver axis dysfunction to persistent liver enzyme abnormalities, nominating these metabolites as potential diagnostic biomarkers and therapeutic targets for HBV-related cirrhosis management (Figures 4a–i).

### Taxonomic and functional composition of the gut microbial community and metabolites was correlated with hepatic abnormalities

#### Gut microbiota-metabolite interactions in hepatic abnormalities

**Figure 5:**
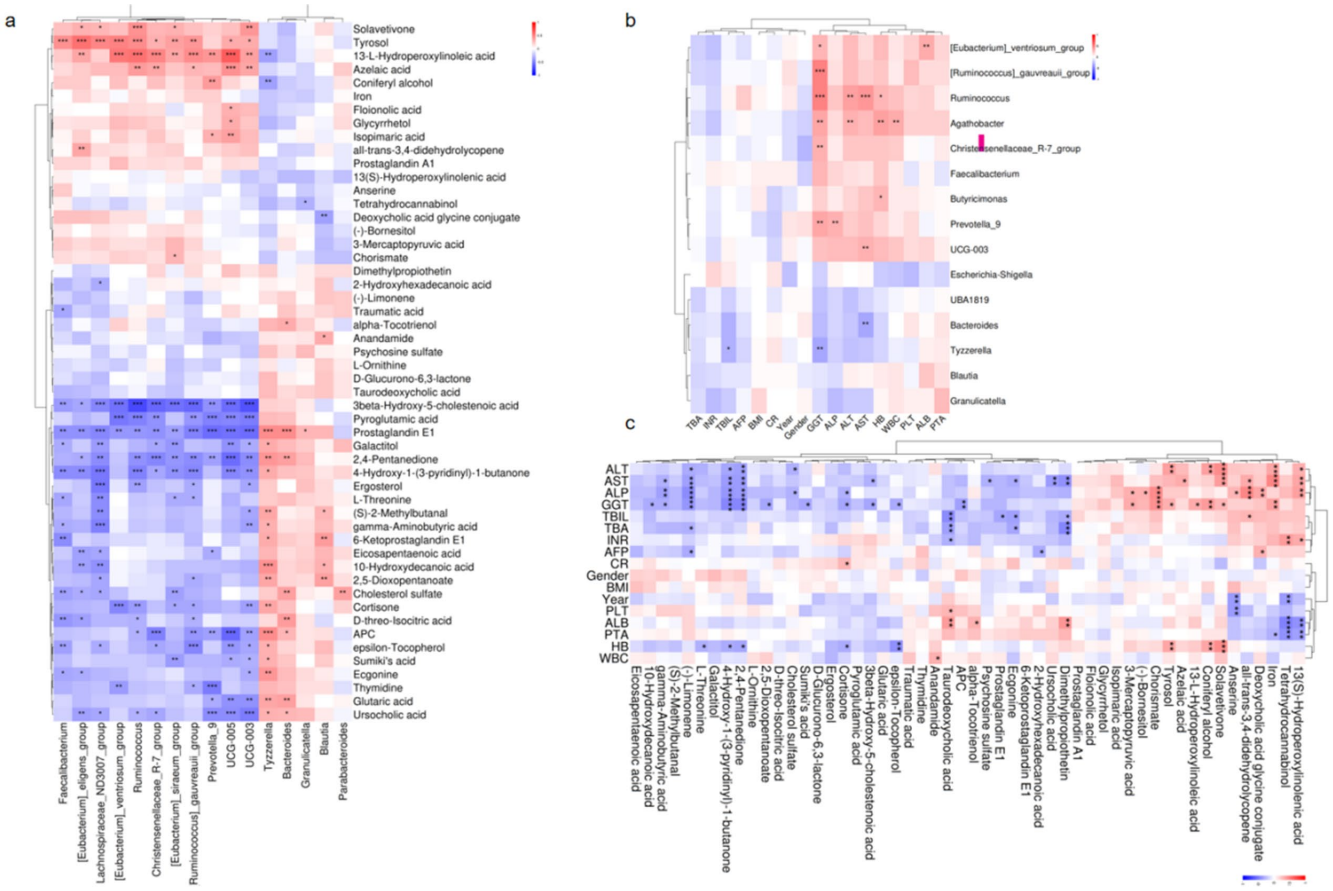
Host-microbiota-metabolite interplay in HBV-related liver cirrhosis. **(a)** Microbial-metabolite correlations: Partial Spearman analysis (*n* = 1,000 bootstrap iterations). **(b)** Microbial-clinical correlations: Partial Spearman analysis (*n* = 1,000 bootstrap iterations). **(c)** Metabolite-clinical correlations: Partial Spearman analysis (*n* = 1,000 bootstrap iterations). *p < 0.05, **p < 0.01, ***p < 0.001.

To explore the interplay between gut microbiota and metabolic dysregulation in HBV-related cirrhosis, we analyzed correlations between microbial taxa and disease-associated metabolites. In patients with persistent liver enzyme abnormalities: The bile acid intermediate 3β-hydroxy-5-cholestenoic acid was negatively correlated with *Ruminococcus* abundance (*r* = −0.711, *p* < 0.001), implicating this genus in bile acid dysregulation. Prostaglandin E1 exhibited positive associations with *Bacteroides* (*r* = 0.246, *p* < 0.001) and *Blautia* (*r* = 0.297, *p* = 0.027) but a negative correlation with *Ruminococcus* (*r* = −0.407, *p* = 0.002), suggesting immunometabolic modulation by these taxa. In patients with normal liver enzymes: Taurodeoxycholic acid levels inversely correlated with *Faecalibacterium* (*r* = −0.287, *p* = 0.033). Ursodeoxycholic acid positively associated with *Bacteroides* (*r* = 0.311, *p* = 0.020) but negatively with *Prevotella_9* (*r* = −0.454, *p* < 0.001). Deoxycholic acid glycine conjugate (DCA-Gly) showed an inverse relationship with *Blautia* (*r* = −0.356, *p* = 0.009). These findings underscore the bidirectional crosstalk between gut microbiota and host metabolism in HBV-related cirrhosis. The identified taxa-metabolite interactions may influence hepatic inflammation and fibrosis progression, offering mechanistic insights into disease pathogenesis (Figure 5a).

#### Microbial and metabolic correlations with clinical parameters

To investigate the association between gut microbiota, microbial metabolites, and hepatic dysfunction, we performed partial Spearman correlation analyses to evaluate potential relationships. The results revealed significant correlations between specific gut microbial taxa and clinical liver function parameters. Notably, elevated alkaline phosphatase (ALP) levels were positively associated with the abundance of *Prevotella_9* (*r* = 0.325, *p* = 0.008) and *Ruminococcus* (*r* = 0.247, *p* = 0.046). Similarly, aspartate aminotransferase (AST) levels exhibited a strong positive correlation with *Ruminococcus* (*r* = 0.431, *p* = 0.0003) but a negative correlation with *Bacteroides* (*r* = −0.350, *p* = 0.004). Furthermore, gamma-glutamyl transferase (GGT) levels were inversely correlated with Tyzzerella (*r* = −0.348, *p* < 0.05) and *Bacteroides* (*r* = −0.260, *p* < 0.05). These findings suggest that gut microbial composition, particularly the relative abundance of *Prevotella_9*, *Ruminococcus*, *Bacteroides*, and *Tyzzerella*, may play a critical role in modulating liver enzyme levels. The observed correlations highlight the potential influence of gut microbiota on hepatic function recovery following antiviral therapy, underscoring the importance of microbial-host interactions in liver-related metabolic processes (Figure 5b).

Key metabolite-clinical correlations revealed several significant associations:(−)-Limonene showed consistent negative correlations with elevated liver enzymes including ALT (*r* = −0.321 to −0.448, *p* < 0.05), AST, GGT, ALP, and total bile acids (TBA). The sterol metabolite 3β-hydroxy-5-cholestenoic acid demonstrated inverse relationships with GGT (*r* = −0.343, *p* = 0.010) and AST (*r* = −0.305, *p* = 0.023). Taurodeoxycholic acid (TDC) was negatively associated with both total bilirubin (TBIL; *r* = −0.436, *p* = 0.0008) and TBA (*r* = −0.372, *p* = 0.005), while γ-aminobutyric acid (GABA) exhibited negative correlations across multiple hepatic parameters (ALP, GGT, AST, and TBA; *r* = −0.367 to −0.287, *p* < 0.05). Notably, distinct patterns emerged for pro-inflammatory metabolites: 13(S)-Hydroperoxylinolenic acid displayed positive correlations with ALT (*r* = 0.316, *p* = 0.022), AST (*r* = 0.355, *p* = 0.010), and ALP (*r* = 0.381, *p* = 0.005). Similarly, the bile acid derivative deoxycholic acid glycine conjugate showed positive associations with ALP (*r* = 0.356, *p* = 0.010) and TBIL (*r* = 0.288, *p* = 0.038). Clinical implications: The significant reduction of bile acid and fatty acid metabolites in patients with hepatic dysfunction (*p* < 0.05) suggests their potential involvement in liver pathology, possibly through gut-liver axis metabolic crosstalk. These findings highlight specific microbial metabolites that may serve as biomarkers or therapeutic targets for hepatic disorders (Figure 5c).

This study demonstrates that specific metabolites, including taurodeoxycholic acid and ursocholic acid, are significantly correlated with multiple microbial genera in patients with abnormal liver enzymes. Elevated levels of ALT, AST, and GGT were negatively associated with reduced levels of key metabolites, underscoring the intricate relationship between gut microbiota, metabolites, and liver enzymes. These findings emphasize the role of microbial diversity and metabolic perturbations in liver recovery, providing potential biomarkers and therapeutic targets for HBV-related cirrhosis management.

## Discussion

Contrary to classical dysbiosis paradigms, virologically suppressed HBV-cirrhosis patients with persistent liver enzyme abnormalities exhibit elevated alpha diversity (Chao1, Shannon, and Simpson indices). We propose this reflects a metabolically driven maladaptive state characterized by: Antiviral therapy-induced niche remodeling, promoting taxonomic diversification without functional restoration; Depletion of hepatoprotective bile acids (UCA/TDC), reducing antimicrobial selection pressure and enabling pathobiont co-existence; Oxidative stress (evidenced by elevated 13(S)-hydroperoxylinolenic acid), favoring resilient but pro-inflammatory taxa (e.g., *Ruminococcus*). Despite higher diversity, functional impoverishment is evident—reduced SCFA producers (*Blautia*) and enriched TLR4 agonists (*Escherichia-Shigella*)—while beta-diversity confirms distinct microbial clustering (ANOSIM *R* = 0.177, *p* = 0.0049). These findings suggest alpha diversity may serve as a context-dependent biomarker in cirrhosis: elevated values during virological suppression could indicate compensatory but unstable microbial reorganization rather than restored health. Therapeutic strategies should prioritize functional restoration (e.g., bile acid supplementation, antioxidant therapy) over mere diversity modulation. Our observations contrast with classical cirrhosis-associated dysbiosis patterns (Bajaj et al., 2014) but align with recent reports of similar diversity paradoxes in metabolic dysfunction-associated steatotic liver disease (Zhang et al., 2021). This implies a conserved microbial stress response to hepatic metabolic disturbances, potentially independent of disease etiology. The co-enrichment of commensal (*Faecalibacterium*) and pathogenic (*Ruminococcus*) taxa in our cohort mirrors post-antiviral therapy studies (Chen et al., 2016), supporting the notion of incomplete microbial recovery despite virological control. In patients with normal liver enzymes, the milder dysbiosis (e.g., *Bacteroides* dominance, higher *Blautia* abundance) may eflect: Compensatory metabolic adaptation via SCFA-mediated immunomodulation (Wang et al., 2024); Antiviral therapy off-target effects on specific taxa (Benítez-Páez et al., 2020); Diagnostic latency, where microbial shifts precede biochemical abnormalities (Boursier et al., 2016). This dissociation between microbial (Benítez-Páez et al., 2020) and biochemical profiles underscores the need for multi-parameter assessments in cirrhosis management.

Our study delineates distinct gut microbiota and metabolite profiles in HBV-related cirrhosis patients achieving virological suppression (undetectable HBV-DNA), stratified by the presence or absence of persistent liver enzyme abnormalities. Key findings reveal a dichotomy between the two cohorts: The normal liver enzyme group exhibited dominance of Bacteroides and Blautia—taxa associated with SCFA production and immunomodulation—alongside enrichment of Escherichia-Shigella, a potential indicator of subclinical inflammation. In contrast, the abnormal liver enzyme group demonstrated marked enrichment of Faecalibacterium, Christensenellaceae_R-7_group, and Ruminococcus, suggesting a dysbiotic state that paradoxically combines commensal resilience with pro-inflammatory potential. The identification of 52 differentially abundant metabolites—including bile acids (e.g., UCA/TDCA), sterols, and oxidative stress markers—highlights two critical pathways: Bile acid disruption: Depletion of hepatoprotective bile acids correlates with impaired microbial conversion capacity, exacerbating cholestatic injury. Oxidative stress: Elevated lipid peroxidation products align with the enrichment of oxidative stress-resistant taxa (e.g., Prevotella_9), creating a feed-forward loop of inflammation. These findings challenge the paradigm that virological suppression universally resolves cirrhosis-associated dysbiosis. Instead, we propose: Persistent enzyme abnormalities reflect functional microbiota disruption beyond taxonomic shifts, evidenced by metabolite-microbe networks (e.g., Ruminococcus-bile acid associations). Therapeutic strategies should target metabolite restitution (e.g., bile acid supplementation) and oxidative stress mitigation to break the inflammation-dysbiosis cycle. The coexistence of beneficial (Faecalibacterium) and pathogenic (Ruminococcus) taxa in the abnormal group mirrors findings in metabolic dysfunction-associated steatotic liver disease, suggesting shared mechanisms of metabolic dysregulation post-viral suppression. Notably, the *Bacteroides*-dominant profile in the normal group resembles microbial recovery patterns observed in NAFLD remission, hinting at potential diagnostic or prognostic utility. While our metabolomics data robustly link specific microbes to functional outcomes, longitudinal studies are needed to establish causality. Additionally, the therapeutic potential of targeting identified metabolites (e.g., FXR agonists for bile acid restoration) warrants clinical validation.

In patients with undetectable HBV-DNA levels and normal liver enzymes, *Bacteroides*—a major producer of propionate, a short-chain fatty acid (SCFA) with anti-inflammatory, anti-lipogenic, and immunomodulatory properties—may contribute to maintaining metabolic and immune homeostasis (Zhang et al., 2025). Propionate-mediated signaling plays a critical role in regulating mucosal immunity, appetite, and cholesterol metabolism, and its deficiency is linked to chronic inflammatory and metabolic disorders, including type 2 diabetes, obesity, and colorectal cancer. Dietary interventions aimed at enhancing colonic propionate production represent a promising strategy for restoring immune and metabolic balance (Wang et al., 2024). The paradoxical elevation of Blautia, another SCFA-producing genus, may reflect compensatory mechanisms to counteract endotoxin-induced damage, as its abundance inversely correlates with portal hypertension severity (Bajaj et al., 2014). While some studies associate Blautia with metabolic disorders such as obesity and type 2 diabetes (Slyepchenko et al., 2017), others suggest its beneficial role in reducing inflammation, as evidenced by its negative correlation with fecal TNF-α levels in normal-weight children. This discrepancy highlights the context-dependent nature of Blautia’s role in host physiology (Benítez-Páez et al., 2020; Slyepchenko et al., 2017; Kashtanova et al., 2018). Conversely, the increased presence of *Escherichia-Shigella*, a well-established driver of TLR4-mediated pro-inflammatory responses, may indicate underlying gut dysbiosis, exacerbating liver injury in chronic hepatitis B (Duan et al., 2019), Similarly, *Prevotella_9*, enriched in patients with progressive hepatocellular carcinoma (HCC), is associated with tumor advancement and poorer clinical outcomes. Although *Faecalibacterium* is generally regarded as anti-inflammatory, its increased abundance in certain chronic liver diseases may paradoxically correlate with disease progression, potentially reflecting the host’s attempt to modulate immune responses in the face of ongoing liver injury (Lee et al., 2022). Although *Faecalibacterium* is generally regarded as an anti-inflammatory bacterium, its increased abundance in certain chronic liver diseases may paradoxically correlate with disease progression, potentially reflecting the host’s attempt to modulate immune responses in the face of ongoing liver injury.

UCA and TDC were significantly reduced in the abnormal group, highlighting impaired bile acid homeostasis as a key feature of persistent liver enzyme abnormalities in HBV-related cirrhosis. UCA, a tertiary bile acid with hepatoprotective and anti-inflammatory properties, plays a crucial role in reducing hepatocyte apoptosis, modulating bile acid metabolism, and alleviating endoplasmic reticulum stress (Beuers et al., 2015). Recent studies further suggest that UCA exerts anti-fibrotic effects by inhibiting hepatic stellate cell activation, positioning it as a potential therapeutic agent for liver fibrosis and cirrhosis. Similarly, TDC, a conjugated bile acid essential for lipid emulsification, contributes to maintaining bile acid balance and reducing hepatic inflammation. The depletion of these protective bile acids underscores the role of bile acid dysregulation in driving liver injury. Dietary supplementation with bile acids, such as UCA, has been shown to reduce experimentally induced hepatic carcinogenesis in animal models, suggesting its potential for clinical translation (Fiorucci et al., 2021). Elevated DCA-Gly and 13(S)-hydroperoxylinolenic acid in HBV-related cirrhosis patients with undetectable HBV-DNA yet persistent liver dysfunction suggest distinct pathophysiological contributions. DCA-Gly accumulation may reflect impaired bile acid detoxification and gut-liver axis disruption, exacerbating hepatocyte injury via mitochondrial dysfunction (Beuers et al., 2015). 13(S)-hydroperoxylinolenic acid, a lipid peroxidation marker, indicates oxidative stress-driven inflammation, potentially activating pro-fibrotic pathways (Kühn and O'Donnell, 2006). These findings underscore metabolic dysregulation as a key driver of liver injury and suggest therapeutic targets, such as bile acid supplementation and antioxidant therapy, to restore metabolic balance and mitigate liver damage.

Our study has several limitations that warrant consideration. The relatively small sample size in microbiome and metabolomic analyses may limit the generalizability of our findings, necessitating validation in larger, multi-center prospective cohorts. Additionally, the use of 16S rRNA sequencing, while informative, provides only genus-level resolution, which may lead to inconsistent taxonomic assignments, as exemplified by the ambiguous abundance patterns of Escherichia-Shigella. To achieve species-level precision and functional insights, shotgun metagenomic sequencing is recommended for future studies. Furthermore, although non-targeted metabolomics offers a comprehensive overview of metabolic perturbations, it lacks the ability to establish causal relationships between microbiome composition and liver disease progression. Functional validation through *in vitro* and *in vivo* models is essential to elucidate these mechanisms. As a cross-sectional study, our design precludes the assessment of temporal changes in the gut microbiome and fecal metabolome, which are critical for understanding the dynamic progression of cirrhosis. Longitudinal studies incorporating serial fecal sampling at different disease stages are needed to identify robust microbiome and metabolite biomarkers associated with disease progression. Addressing these limitations through advanced sequencing technologies, larger cohort studies, and mechanistic investigations will significantly enhance our understanding of the gut-liver axis and inform the development of targeted therapeutic strategies for HBV-related cirrhosis.

## Conclusion

In summary, this study demonstrates that gut microbiota dysbiosis and metabolic perturbations significantly influence persistent liver enzyme abnormalities in cirrhotic patients achieving HBV-DNA undetectability. The marked depletion of anti-inflammatory genera (e.g., Blautia) and hepatoprotective bile acids (e.g., ursocholic acid, UCA) correlates positively with hepatic dysfunction. This bidirectional gut-liver crosstalk manifests as: Progressive liver injury exacerbating intestinal ecological imbalance, and gut-derived metabolites accelerating disease progression through systemic inflammation. Notably, we propose the gut-liver axis remains a central metabolic regulator even post-virological control, with its associated microbial signatures and metabolite profiles serving as non-invasive prognostic tools. These findings establish a mechanistic foundation for targeted interventions including microbiota modulation and metabolite supplementation. Future investigations should employ longitudinal cohorts with serial fecal sampling across cirrhosis stages to dynamically track progression-associated biomarkers. Integrated multi-omics approaches, expanded cohorts, and mechanistic validation will further elucidate gut-liver interactions, advancing precision therapeutics for HBV-related cirrhosis.

## Data Availability

The data presented in this study are deposited in the NCBI Sequence Read Archive (SRA) repository, accession number PRJNA1309012.

## Footnotes

^1^ http://www.hmdb.ca

^2^ https://www.genome.jp/kegg/
